# Hypothalamic metabolic compartmentation during appetite regulation as revealed by magnetic resonance imaging and spectroscopy methods

**DOI:** 10.3389/fnene.2013.00006

**Published:** 2013-06-13

**Authors:** Blanca Lizarbe, Ania Benitez, Gerardo A. Peláez Brioso, Manuel Sánchez-Montañés, Pilar López-Larrubia, Paloma Ballesteros, Sebastián Cerdán

**Affiliations:** ^1^Department of Experimental Models of Human diseases, Laboratory of Imaging and Spectroscopy by Magnetic Resonance, Instituto de Investigaciones Biomédicas “Alberto Sols” CSIC/UAMMadrid, Spain; ^2^Departmento de Informática, Escuela Politécnica Superior, Universidad Autónoma de MadridCantoblanco, Madrid, Spain; ^3^Laboratorio de Síntesis Orgánica e Imagen Molecular por Resonancia Magnética, Facultad de Ciencias, Universidad Nacional de Educación a Distancia, Unidad Asociada al CSICMadrid, Spain

**Keywords:** appetite regulation, hypothalamus, neuroendocrine signaling, neuroglial compartmentation, magnetic resonance imaging, magnetic resonance spectroscopy

## Abstract

We review the role of neuroglial compartmentation and transcellular neurotransmitter cycling during hypothalamic appetite regulation as detected by Magnetic Resonance Imaging (MRI) and Spectroscopy (MRS) methods. We address first the neurochemical basis of neuroendocrine regulation in the hypothalamus and the orexigenic and anorexigenic feed-back loops that control appetite. Then we examine the main MRI and MRS strategies that have been used to investigate appetite regulation. Manganese-enhanced magnetic resonance imaging (MEMRI), Blood oxygenation level-dependent contrast (BOLD), and Diffusion-weighted magnetic resonance imaging (DWI) have revealed Mn^2+^ accumulations, augmented oxygen consumptions, and astrocytic swelling in the hypothalamus under fasting conditions, respectively. High field ^1^H magnetic resonance *in vivo*, showed increased hypothalamic myo-inositol concentrations as compared to other cerebral structures. ^1^H and ^13^C high resolution magic angle spinning (HRMAS) revealed increased neuroglial oxidative and glycolytic metabolism, as well as increased hypothalamic glutamatergic and GABAergic neurotransmissions under orexigenic stimulation. We propose here an integrative interpretation of all these findings suggesting that the neuroendocrine regulation of appetite is supported by important ionic and metabolic transcellular fluxes which begin at the tripartite orexigenic clefts and become extended spatially in the hypothalamus through astrocytic networks becoming eventually MRI and MRS detectable.

The hypothalamus is a small cerebral structure responsible for the integral homeostasis of vital systemic functions including global energy metabolism, appetite, thirst and osmoregulation, thermoregulation, circadian rhythms, and some fundamental survival responses such as aggressiveness (Swaab et al., 1992; Ganong, 1993; Lin et al., 2011). It operates as a highly sophisticated neuroendocrine transducer, sensing peripheral endocrine signals and transforming them in intracerebral excitatory or inhibitory neurotransmitter events that deliver the homeostatic response back to the periphery (McEwen, 1989; Levin et al., 2011). Hypothalamic function involves frequently the operation of highly elaborated feed-back control loops (Figure 1).

**Figure 1:**
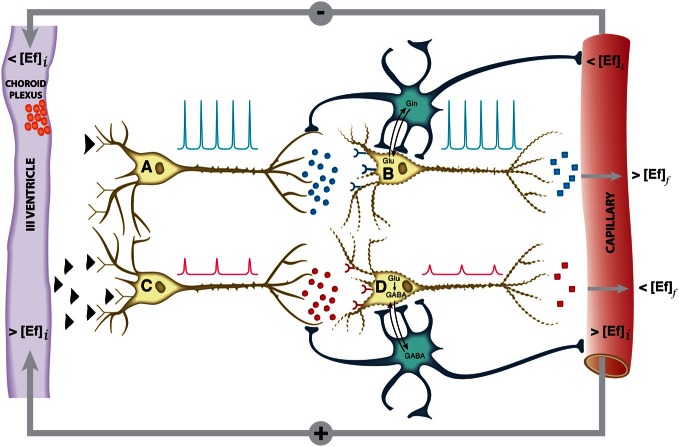
**Schematic representation of the neuroendocrine interface and feed-back control loops**. Neuroendocrine integration may involve both, neurons receiving endocrine signals and releasing neurotransmitters (A, C) or neurons receiving neurotransmitter signals and releasing neuropeptides or hormones (B, D). A typical feed-back control loop is illustrated. The initial decrease in plasma concentration of an endocrine inhibitory effector (<[Ef]_*i*_), passes the blood brain barrier, releasing the inhibition of neuron A and triggering action potentials (blue spikes) and the synaptic release of an excitatory neurotransmitter as glutamate (blue circles). This activates neuron B and triggers the release of an endocrine signal resulting eventually in an increase in the final plasma concentration of the effector (>[Ef]_*f*_). Glutamatergic neurotransmission involves necessarily the operation of the neuroglial glutamine cycle. The initial increase in the plasma concentration of the effector (>[Ef]_*i*_) inhibits neuron C, decreasing the frequency and amplitude of the action potentials and triggering the release of an inhibitory neurotransmitter as GABA (red circles). This causes neuron D to reduce or suspend the release of the effector Ef, resulting eventually in an homeostatic reduction in the plasma concentration of (<[Ef]_*f*_),. GABAergic neurotransmission involves necessarily the neuroglial glutamate-GABA cycle. Both negative and positive feed-back loops tend to maintain the plasma concentration of the effector Ef stable within a narrow range. Glu, glutamate; Gln, glutamine; GABA, γ-aminobutyric acid.

Activation of the hypothalamic interface is thought to proceed essentially in two steps, an initial endocrine activation, involving the receptor-mediated interaction of the hormone or neuropeptide with the presynaptic terminal, followed either by the activation or inhibition of excitatory or inhibitory neurotransmitter release at the postsynaptic cleft. The neurotransmitters glutamate and GABA play a central role in this process mediating glutamatergic (Tong et al., 2007) or GABAergic (Xu et al., 2012) neurotransmissions through specific neuronal pathways of the hypothalamus. These pathways trigger eventually the release of hypothalamic neuropeptides or hormones to the blood stream, to other hypothalamic structures and the hypophysis (McEwen, 1989; Thorburn and Proietto, 1998) or activate the brain stem autonomic neurocircuitry (Palkovits, 1999), all these events geared to maintain systemic homeostasis.

In the last decades, important progress has been reached characterizing the initial endocrine step, identifying the corresponding systemic and intrahypothalamic neuropeptides and receptors or the morphological pathways and tracts transmitting these signals within the different hypothalamic nuclei or even to extra-hypothalamic structures (McEwen, 1989; Lantos et al., 1995). Progress has been slower, however, in the characterization of the neurotransmitter events underlying the neuroendocrine response. In this respect, early interpretations conceived the hypothalamic response as a neuronal only event. The evolution of the tripartite synapse concept (Araque et al., 1999; Halassa et al., 2007; Santello et al., 2012), however, revealed the essential role of astroglia in the modulation of synaptic neurotransmission, gaining for astrocytes a fundamental role in neuroendocrine signaling (Garcia-Ovejero et al., 2005; Garcia-Segura et al., 2008). In some important cases, as in thyroid hormones, astrocytes are even required not only to modulate synaptic transmission, but to generate the active endocrine response (Mohacsik et al., 2011). Furthermore, glutamatergic or GABAergic neurotransmissions involve necessarily the operation of transcellular cycles of glutamate and GABA between neurons and astrocytes (Hertz, 2004; Dienel and Hertz, 2005), stressing the fundamental role of neuroglial compartmentation in hypothalamic function. However, further improvements in our understanding of the role of metabolic compartmentation in neuroendocrine function have been often hampered by the limited accessibility of sufficiently robust non-invasive methods to monitor neuronal activation and transcellular neurotransmitter cycling in the hypothalamus *in vivo*.

Magnetic Resonance Imaging (MRI) and Spectroscopy (MRS) approaches are known to be well endowed to observe hypothalamic morphology and function. Briefly, Manganese-enhanced MRI (MEMRI) techniques allow to monitor neuronal activation through the accumulation of Mn^2+^ and its effects in T_1_-weighted images (Koretsky and Silva, 2004), Blood Oxygen Level-dependent (BOLD) methods detect cerebral activation through associated hemoglobin deoxygenation and perfusion changes (Zhu et al., 1998; Logothetis and Wandell, 2004) and Diffusion-weighted Imaging (DWI) visualize microstructural changes in the diffusion coefficient of water (Le Bihan, 2003), reflecting most probably activation induced neurocellular swelling events. In addition, ^1^H MRS *in vivo* is able to characterize the metabolic profile of the hypothalamus and its changes during activation (Duarte et al., 2012) and ^13^C MRS provides comprehensive information on neuroglial oxidative metabolism and neurotransmitter cycling (Cruz and Cerdan, 1999; Gruetter et al., 2003; Rothman et al., 2003). Taken together, these techniques are beginning to yield precious information on hypothalamic physiology. However, a critical overview of the information gained with the different methods and an integrative interpretation of the results obtained becomes currently necessary, to be able to recapitulate and design more precisely the protocols of future strategies.

In this review, we examine the information gained thus far with these approaches and provide an integrative interpretation that highlights the vital role of hypothalamic neuroglial compartmentation in the cerebral control of global energy homeostasis *in vivo*.

## Hypothalamic control of appetite

Recent years have witnessed an important development in the understanding of the hypothalamic mechanisms involved in appetite control and global energy homeostasis (Coll et al., 2007). Appetite control is currently understood to operate on the balance of positive and negative peripheral signals from the adipose tissue, the pancreas, and the gastrointestinal tract, modulating intrahypothalamic and brain stem autonomic activities that determine the early hunger or satiety responses and the long-term body weight and energy balance (Schwartz and Morton, 2002; Morton et al., 2006). Peripheral signals from the gut include mainly peptide YY (PYY), oxyntomodulin (OXM), ghrelin, glucagon-like peptide 1 (GLP-1), and colecystokinin (CCK). Adipose tissue, pancreatic, and gut-derived peptides influence the hypothalamic circuitry providing short-term hunger or satiety signals and resulting in the long term in anabolic (−) or catabolic (+) effects in energy expenditure, increasing or decreasing body weight. In particular, these mediators modulate the activation of the arcuate (ARC), paraventricular (PVN), dorsomedial nuclei (DMN), and ventromedial nuclei (VMN) of the hypothalamus which control food intake through a delicate balance of orexigenic and anorexigenic pathways operated by specific neurons and neuropeptides (Stanley et al., 2005).

**Figure 2:**
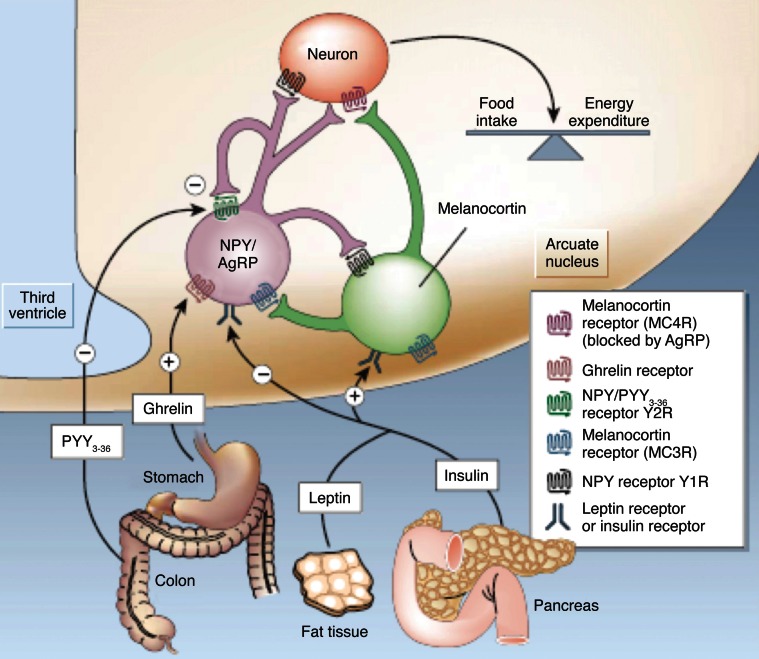
**Hypothalamic control of global energy balance**. Appetite is regulated by a complex feed-back loop involving endocrine signals originated in peripheral tissues and intrahypothalamic peptides. Leptin and insulin inhibit the orexigenic NPY/AgRP neurons (purple) and stimulate the anorexigenic melanocortin neurons (green), resulting in a reduction of food intake. Ghrelin or PYY_3–36_ activate or inhibit the NPY/AgRP neurons resulting in orexigenic or anorexigenic responses, respectively. Taken from Schwartz and Morton (2002). Reproduced with permission of the publisher.

Figure 2 illustrates our current views on the mechanism of appetite control within the hypothalamus. Hypothalamic control of energy homeostasis involves the modulation of orexigenic (stimulation of food intake) and anorexigenic (satiety signals) pathways, that determine the positive or negative balance between food intake and energy expenditure (Schwartz et al., 2000; Schwartz and Morton, 2002; Morton et al., 2006). Briefly, leptin and insulin produced by fat tissues and pancreas, circulate in blood in amounts proportional to body fat and blood glucose. These long-term systemic effectors reach easily the hypothalamic ARC nucleus, an area of relatively permeable Blood Brain Barrier (BBB) and thus highly accessible to activation by systemic effectors. Insulin and leptin, inhibit the orexigenic Neuropeptide Y (NPY) and Agouti-related Peptide (AgRP) neurons (purple) and activate the anorexigenic neurons (green) of the melanocortin (α-MSH)/cocaine and amphetamine-regulated transcript (CART) pathways, resulting in decreased food intake and increased energy expenditure. Long-term increases in leptin or insulin lead to receptor desensitization and insulin or leptin “resistance” increasing plasma glucose levels and fat accumulation, producing eventually obesity and diabetes. Ghrelin and peptide PYY_3−36_, released by the stomach and the colon, respectively, provide the Arcuate with positive or negative short-term signals of appetite or satiety through the selective activation or inhibition of the NPY/AgRP neurons, resulting in hunger or satiety, respectively (Tang-Christensen et al., 2004).

Despite these important advances in the understanding of the endocrine processes controlling food intake and energy expenditure, less is known on how these modify the neuroglial metabolic coupling mechanisms supporting the activation or inhibition of the orexigenic or anorexigenic pathways. However, important evidence supports the crucial role of glutamatergic or GABAergic neurotransmissions on hypothalamic function (Collin et al., 2003; Hentges et al., 2004). In particular, intracerebral glutamate administration is known to elicit an intense orexigenic response (Stanley et al., 1993) while knock out mice in glutamate or GABA vesicular transporters are known to exhibit altered feeding behavior (Tong et al., 2007; Xu et al., 2012). Notably, how glutamatergic or GABAergic neurotransmissions are modulated by orexigenic or anorexigenic stimuli *in vivo* has not been directly addressed.

## Magnetic resonance imaging studies of hypothalamic appetite regulation

### Manganese-enhanced magnetic resonance imaging (MEMRI)

MEMRI is currently thought to directly reflect the neuronal accumulation of Mn^2+^ through Voltage-dependent Calcium Channels in stimulated brain areas, an event that extends transynaptically and enables MEMRI to map neuronal connectivities (Pautler, 2004). Mn^2+^ accumulation may actually exceed the neuronal tracts and extend to surrounding astrocytes and astrocytic networks, since abundant gap junctions exist in astrocytes (Andrew et al., 1981) and neuronal activation has shown to elicit astrocytic intracellular and intercellular Ca^2+^ waves (Jaffe, 2006, 2008, 2010). Paramagnetic Mn^2+^ ions mimic closely the size of diamagnetic Ca^2+^, thus providing an ideal surrogate probe to monitor Ca^2+^ dynamics during neuronal activation. Hydrated Mn^2+^ ions are classically known to induce a strong reduction in the T_1_ of water, resulting in bright contrast in T_1_-weighted images in those activated areas accumulating Mn^2+^ (Lee et al., 2005). MEMRI is not devoid from limitations, since Mn^2+^ administration is known to become neurotoxic, competing with endogenous Ca^2+^ fluxes, pertubing hypothalamic levels of metabolites and interfering with the operation of vital metabolic pathways as the tricarboxylic acid cycle and neurotransmitter cycles (Zwingmann et al., 2003, 2004).

Despite these limitations, MEMRI has been successfully used to detect brain activity (Aoki et al., 2002) and neuronal architecture (Aoki et al., 2004) in rodents since the early 2000s, when the first application to the study of hypothalamic functionality appeared (Morita et al., 2002). Morita et al. detected increases in T_1_-weighted images in the rat brain after intravenously infusing MnCl_2_, but they had to disrupt the BBB for manganese to diffuse properly through the brain. As an activation agent, they injected NaCl to the rat brain and found signal increases in areas involved in central osmotic regulation, including the hypothalamic area. Their observations were validated by a positive correlation with c-Fos expression levels in the activated areas and demonstrated, for the first time to our knowledge, the possibility of studying hypothalamic functionality with Mn^2+^-enhanced MRI, although this required BBB disruption. Later on, in 2006, MEMRI was successfully implemented without compromising the BBB (Yu et al., 2005) to map regions of accumulated sound-evoked activity in mice, after intraperitoneal administration of MnCl_2_. Soon after, the first MEMRI study of hypothalamic activation associated with feeding, without compromising the BBB (Kuo et al., 2006) was published. Authors infused intravenously MnCl_2_ during the MRI acquisition protocol and compared signal enhancement in the hypothalamus of fed or overnight-fasted mice, obtaining significant differences in different hypothalamic nuclei. This revealed that region-specific Mn^2+^ enhancement in the mouse brain could be modulated by fasting. Since then, several MEMRI studies have focused on the hypothalamic functionality associated to feeding, by studying the effect of peptide administration and its pathways of activation (Chaudhri et al., 2006; Parkinson et al., 2009; Hankir et al., 2011), cerebral activation in transgenic mice (Delgado et al., 2011) and hypothalamic response to alterations of food intake (Just and Gruetter, 2011; Anastasovska et al., 2012).

Finally, recent years have witnessed growing interest on MEMRI applications, geared to a better understanding of the molecular mechanisms by which Mn^2+^ produces alterations of the hypothalamic physiological processes. In particular, a significant number of publications focused on studies combing MEMRI with other imaging or spectroscopic techniques (Delgado et al., 2011; Just et al., 2011; Gutman et al., 2012), or even using MEMRI information to understand other functional techniques (Silva, 2012).

**Figure 3:**
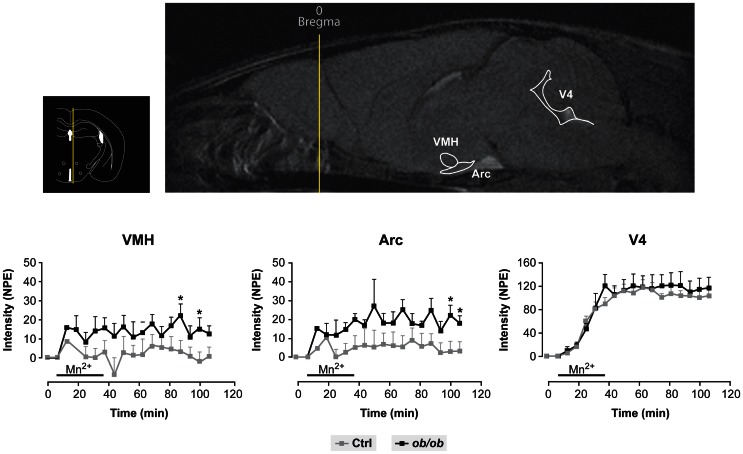
**Representative MEMRI response in obese and control mice**. Inset upper left: Localization of the hypothalamus, third and fourth ventricles in an anatomical atlas (Paxinos and Franklin, 2001). Upper right: Mn^2+^ induced increase in MR signal intensity (MEMRI) in the arcuate nucleus (Arc), ventromedial nucleus (VMH) and the fourth ventricle (V4). Lower panels: Kinetics of MEMRI increase in VMH, Arc and V4 during and after Mn^2+^ infusion. Note that the obese animals (black squares) show a larger increase in MEMRI than the controls (gray squares) in VMH and Arc, but not in V4, revealing a specific obesity effect in these hypothalamic nuclei. Taken from Delgado et al. (2011). Reproduced with permission of the publisher.

Figure 3 provides a useful frame for these concepts illustrating the use of MEMRI in the hypothalamus of control and obese mice. Mn^2+^ infused in the tail vein of normal and obese mice resulted in a larger increase in the intensity of the Arcuate and VMN of obese animals as compared to the controls, a circumstance revealing the orexigenic stimulation of obese animals and the high anatomical and neurophysiological resolution of the approach.

### Blood oxygenation level dependent (BOLD) contrast

BOLD imaging is one of the most widely used techniques to study brain function in animals and man, based on detecting increases in oxygen consumption and associated hemodynamic responses during neuronal activation. In the neuronal activation process, the ratio between deoxyhemoglobin (paramagnetic) and oxyhemoglobin (diamagnetic) changes, and by studying this ratio, BOLD (Ogawa et al., 1990) can successfully map brain activity.

The use of functional neuroimaging in the study of appetite control started in the late nineties, by monitoring the hypothalamic function after glucose uptake in obese or lean humans (Matsuda et al., 1999), demonstrating for the first time the existence of differential hypothalamic function between lean and obese subjects. Almost at the same time, BOLD imaging detected hypothalamic functionality in a rat model following intraperitoneal glucose administration (Mahankali et al., 2000) by recording significant decreases of the MRI signal in the hypothalamic region after the injection. A few years later, a positive correlation between blood-oxygenation-level-dependent (BOLD) contrast fMRI and c-fos protein expression was established in activated areas of the rat brain after the administration of anorexigenic agents (Stark et al., 2006), thus validating the use of BOLD in anesthetized rats to identify the pathways of brain response to appetite-modulating signals. Also in rats, different hypothalamic response to glucose administration was detected in lean and obese animals (Chen et al., 2007), with an attenuated BOLD response in obese rats that was positively correlated with the percentage of positive NPY cells in the hypothalamus, and with significantly lower levels of 5-hydroxytryptamine (5-HT). Since then, the use of BOLD imaging for the study of appetite regulation in animals has generated an important number of contributions, mainly related to the effects on hypothalamic activation after the administration of different diets or peptides to rats (Min et al., 2011; Li et al., 2012) and its correlation with endogenous levels of neuropeptides.

In humans, early fMRI studies started investigating cerebral activation with food pictures (Killgore et al., 2003) and the hypothalamic response to different tastes and calories (Smeets et al., 2005). It soon became clear that appetite in humans was the result of very complex and interrelated neuronal circuits, including not only hypothalamus and brainstem, which are the principal homeostatic brain areas regulating body weight, but also corticolimbic and higher cortical regions. Consequently, different authors investigated the neuronal networks that responded to specific orexigenic or anorexigenic signals (Batterham et al., 2007; Miller et al., 2007; Malik et al., 2008). Currently, the applications of BOLD fMRI on studies of appetite regulation are mainly dedicated to the study of hypothalamic response to glucose (Vidarsdottir et al., 2007; Purnell et al., 2011), to the establishment of differences between fMRI responses in obese and non-obese humans (Tomasi et al., 2009), and to the effects of appetite modulating hormones derived from the gastrointestinal tract and adipose tissue, mainly ghrelin (Jones et al., 2012), insulin (Guthoff et al., 2010) and leptin (Baicy et al., 2007; Farooqi et al., 2007).

**Figure 4:**
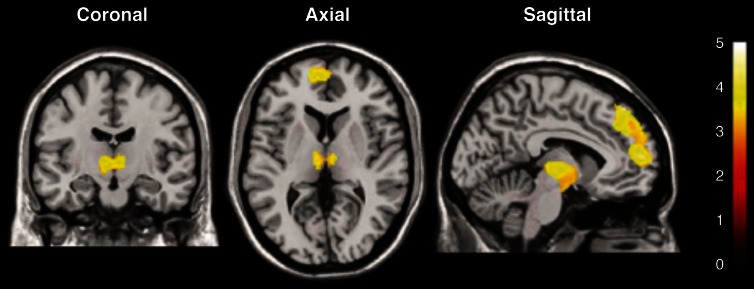
**Statistical parametric maps of brain activated regions in the human brain, as measured by BOLD, during the presentation of high-calorie food images**. The color bar reflects the scale of the SPM statistic used for the analysis. The dorsolateral and medial prefrontal cortex, the thalamus and the hypothalamus showed significant activation (*P* < 0.005) relative to the control pictures of non-edible food related utensils Reproduced from Killgore et al. (2003) with permission of the publisher.

Figure 4 illustrates a representative application of BOLD imaging to appetite regulation in a study that monitored hypothalamic activation in humans, as induced by a paradigm that showed images of high- and low-calorie foods. Briefly, fMRI was applied to investigate cerebral responses of 13 healthy women by presenting three categories of images: high-calorie, low-calorie and non-edible food-related utensils. They found areas of activation common to food stimuli regardless of the calorie content, such as the bilateral amygdala/hippocampus region. Besides, high-calorie food stimuli were found to be associated with significant clusters of activation within the medial and dorsolateral prefrontal cortex, medial dorsal thalamus, hypothalamus, corpus callosum, and cerebellum.

### Diffusion-weighted imaging (DWI)

Diffusion-weighted Imaging (DWI) provides information on the diffusion behavior of water molecules in biological tissues, and can be used to probe and define tissue structures at microscopic scales (Le Bihan, 2003). Since its introduction in 1985, it is the modality of choice for the assessment of stroke in patients (Schellinger et al., 2000) and for studies of white matter diseases (Hagmann et al., 2007).

The interpretation of the biophysical models underlying diffusion changes observed in physiological or pathological states has been a matter of debate in the last decades. Indeed, several models that overcome the conventional monoexponential approach of diffusion (Le Bihan, 2003) have been proposed (Assaf et al., 2002; Jensen and Helpern, 2010; Grinberg et al., 2011).

Conventional DWI has been used to the study of appetite regulation in the hypothalamus (Alkan et al., 2008). This study compared apparent diffusion coefficients (ADC) values in brain regions of obese and non-obese human subjects. Among other areas, the hypothalamus of obese subjects was found to have higher ADC values than the hypothalamic areas of lean patients. Results were explained as an alteration of fluid distribution in obese subjects due to a vasogenic edema. In fact, it has been demonstrated that consumption of fat-rich diets activates proinflammatory responses in the hypothalamus (De Souza et al., 2005), and that, in mice, the ability of high fat diets to induce obesity depends upon the neuronal expression of the mediator of inflammatory signaling MyD88 (Kleinridders et al., 2009). Furthermore, the relationship between hypothalamic inflammation and obesity has become a matter of study and debate (Wisse and Schwartz, 2009; Wang et al., 2012), and DWI is ideally endowed to evaluate its existence *in vivo* (Cazettes et al., 2011). Recent results from our laboratories (Lizarbe et al., 2013) suggest that hypothalamic inflammation may occur not only in obesity, but also transiently during fasting states.

Some years ago, the behavior of diffusion in biological tissues was suggested to represent slow and fast diffusion phases of water that were modified during neuronal activation process (Niendorf et al., 1996; Le Bihan et al., 2006). Since then, several contributions have used the biphasic model to detect and describe more precisely brain activation using DWI in man and studies *in vitro* (Flint et al., 2009; Kohno et al., 2009; Aso et al., 2013). Even model-free approaches have been proposed that confirm and extend the results obtained with the biexponential model (Lizarbe et al., 2013). The use of functional DWI (fDWI) in the study of hypothalamic activation associated to feeding came also from this study. Our results showed that the diffusion coefficients of water in the hypothalamus changed with fasting in both mice and humans. In mice, it became possible to detect changes in individual hypothalamic nuclei, including the ARC, the DMN and the VMN. On these grounds, the application of fDWI to the study of brain activation in general and hypothalamic activity in particular, appears to open a new avenue within the functional neuroimaging field, even in humans. Diffusion changes associated to activation are thought to occur closer -temporally and spatially- to the activated areas than the physiological changes detected with BOLD (Le Bihan et al., 2006), avoiding the use of potentially toxic Mn^+2^ doses. Besides, the possibility of using DWI to detect directional differences through the implementation of Diffusion Tensor Imaging approaches (Le Bihan et al., 2001; Ahn and Lee, 2011) may allow the investigation of neuronal tracts and their potential alterations under different kinds of appetite-related disturbances.

**Figure 5:**
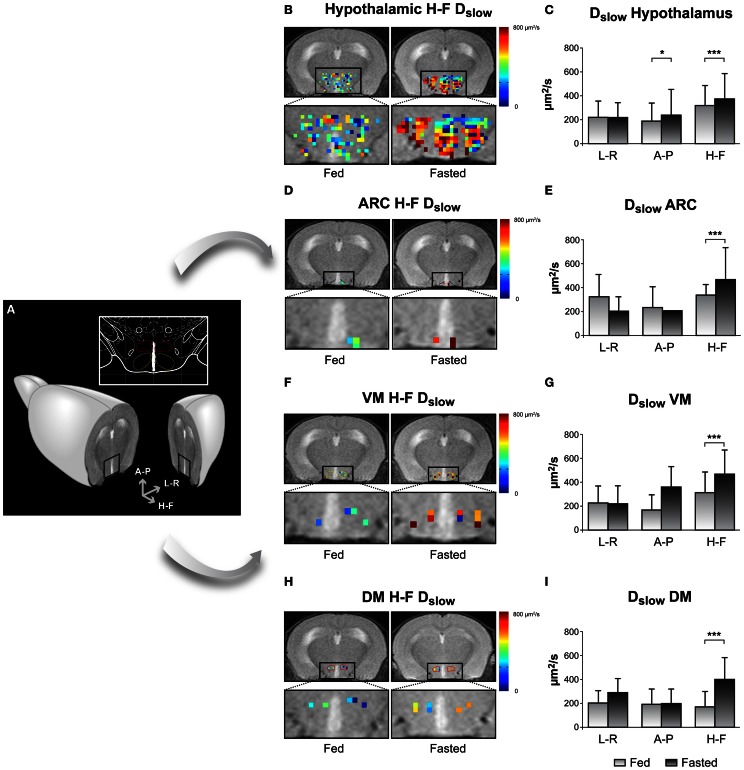
**Imaging appetite by fDWI in the hypothalamic nuclei of the mouse brain. (A)** Axial MRI section containing the hypothalamus in a representative mouse brain and in a brain atlas (inset) showing the localization of the main periventricular hypothalamic nuclei: Dorsomedial Nucleus (DMN, red), Ventromedial Nucleus (VMN, yellow), and Arcuate Nucleus (Arc, blue). **(B,D,F,H)** Hypothalamic color maps of diffusion parameters from fed or fasted mice, superimposed to T_2w_ images in different hypothalamic nuclei. The hypothalamic region is depicted enlarged in the corresponding lower panels. **(C,E,G,I)** D_slow_ bar graphs of parameter values corresponding to the **(B,D,F,H)** panels, respectively. ^*^p < 0.05, ^***^p < 0.001. Reproduced from Lizarbe et al. (2013) with permission of the publisher.

An example on the use of DWI imaging in the study of hypothalamic activation is illustrated in Figure 5, through the changes observed in the diffusion parameters of water in different hypothalamic nuclei including the ARC, VMN, and DMN, depicted in Figure 5A (Lizarbe et al., 2013). Panels **5B**,**D**,**F**, and **H** show parameter maps of the slow diffusion coefficient (D_slow_) of a representative mouse in the fed (left) or overnight-fasted (right) state, in the hypothalamus, ARC, VMN, and DMN nuclei, respectively. Bar graphs in panels **5C,E,G**, and **I** show mean values of the same parameter in six mice. The significant increase of D_slow_ with fasting in all nuclei may be interpreted as the consequence of activation-induced astrocytic swelling.

## Multinuclear magnetic resonance spectroscopy studies of appetite regulation

The use of imaging methods may be complemented by several advanced spectroscopy strategies including mainly ^1^H MRS *in vivo* and *ex vivo* ^1^H and ^13^C HRMAS. These methods have been shown to overcome the need to use large voxel volumes, a limitation precluding earlier the applications of MRS *in vivo* to hypothalamic physiology.

### High field ^1^H MRS *in vivo*

**Figure 6:**
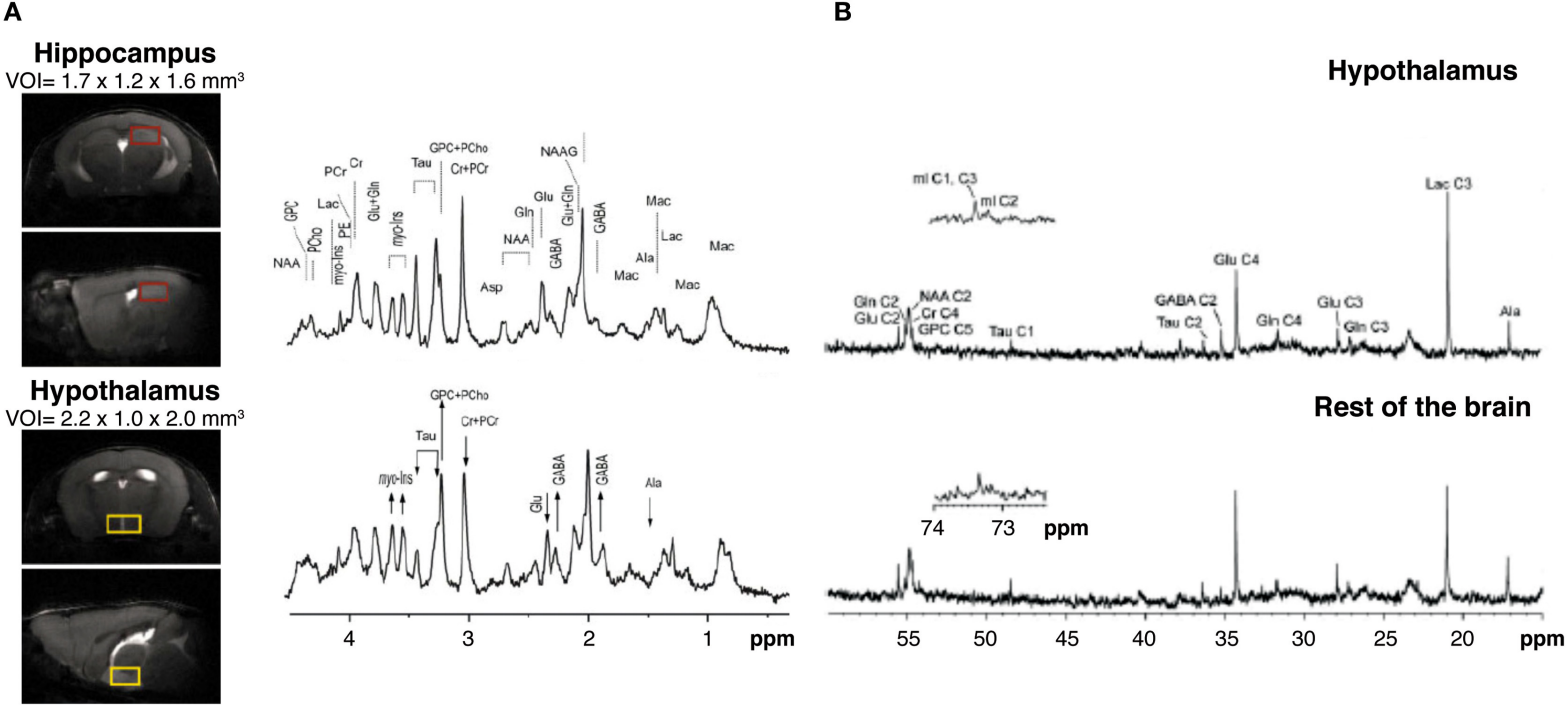
**Multinuclear NMR spectroscopy of the hypothalamus. (A)** ^1^H MRI *in vivo* from selected voxels of mouse hypothalamus (lower panels, yellow region) as compared to the hippocampus (upper panels, red region) and corresponding 1H NMR spectra. Note the relative increases in hypothalamic GABA and myo-inositol (arrow up) and the decreases in glutamate and taurine content (arrow down). Taken from Lei et al. (2010) and reproduced with permission of the publisher. **(B)** Representative ^13^C HRMAS of hypothalamic biopsies (top) prepared after (1-^13^C) glucose infusion as compared to the rest of the brain (bottom) in a fasted mouse. Reproduced from Violante et al. (2009) with permission of the publisher.

High field ^1^H MRS (14.1 Tesla) has been shown to be able to obtain high quality metabolic profiles from the mouse hypothalamus *in vivo* either unilaterally (2.2 μL voxels) or bilaterally (4.4 μL voxels) (Lei et al., 2010; Duarte et al., 2012). Authors reported that the metabolic profile of the hypothalamus is different from other cerebral structures as the hippocampus, containing larger concentration of γ-aminobutyric acid and myo-inositol and lower concentrations of taurine (Figure 6A). High field ^1^H MRS was used to characterize the effects of Mn^2+^ in the metabolic profile of the rat hypothalamus under the paradigm of Dehydration-induced Anorexia (DIA) (Just and Gruetter, 2011). Results showed that γ-aminobutyric acid had an essential role in the maintenance of energy homeostasis in the hypothalamus, independently of the condition investigated. Glutamate, glutamine, and taurine, however, appeared to respond more accurately to Mn^2+^ exposure. When comparing DIA and overnight fasting, GABA levels increased in both, but lactate increased significantly only in DIA. Taken together, these studies showed that high field ^1^H MRS *in vivo* coupled with MEMRI, could provide very relevant information on the hypothalamic mechanisms involved in the control of food intake, global energy balance, and body weight control in rodents.

### ^13^C and ^1^H high resolution magic angle spinning spectroscopy

^13^C Magnetic Resonance Spectroscopy is a method that has shown previously an enormous potential in the investigation of neuroglial coupling mechanisms, both *in vivo* and *in vitro* (Cruz and Cerdan, 1999; Gruetter et al., 2003; Rothman et al., 2003; Rodrigues et al., 2009). However, the low natural abundance of ^13^C (1.1%) and the reduced sensitivity of the method, imposed the use of relatively large voxel sizes *in vivo*, exceeding significantly the dimensions of the hypothalamus. To overcome this, a novel collection of High Resolution Magic Angle Spinning (HRMAS) ^13^C methods *ex vivo* were implemented recently. By acquiring NMR spectra of biopsy samples, inclined 54.7° with respect to the static magnetic field, the dipolar couplings that broaden the resonances *in vivo* are removed and high resolution spectra similar to those obtained in solution, can be obtained from samples as small as 5–10 mg, a size comparable with that of the rodent brain hypothalamus. Using this technology, authors investigated (Figure 6B) the effects of overnight fasting and ghrelin administration on the metabolic profile and the incorporation of ^13^C from (1-^13^C) glucose into hypothalamic metabolites (Violante et al., 2009). Overnight fasting induced significant increases in ^13^C incoporation into (2-^13^C) GABA and (3-^13^C) lactate, while the infusion of the orexigenic peptide ghrelin did not affect ^13^C labeling in these metabolites. These results revealed that overnight fasting appears to increase GABAergic neurotrasmission and glycolysis, but additional factors other than ghrelin were required to elicit this complex hypothalamic response.

The neuroglial mechanisms underlying leptin signaling in the hypothalamus were recently investigated in control and ob/ob mice, combining MEMRI with ^1^H and ^13^C HRMAS and infusions of (1-^13^C) glucose, a primarily neuronal substrate or (2-^13^C) acetate, a predominantly glial substrate (Delgado et al., 2011). Leptin defficient obese mice showed increased MEMRI contrast in the ARC and VMN (Figure 3) and augmented ^13^C accumulation in the hypothalamic glutamate and glutamine carbons from (1-^13^C) glucose, but not from ^13^C acetate. Together, this evidence showed for the first time that the increased MEMRI effect associated to neuronal activation of the orexigenic pathways in the obese mice was accompanied by increased oxidative metabolism and glutamate-glutamine cycling. Together with earlier ^13^C HRMAS evidences on increased GABAergic performance after overnight fasting, the picture emerges that orexigenic stimulation results in increased glutamatergic and GABAergic neurotransmission, implying augmented transcellular cycling of glutamate-glutamine and GABA between neurons and astrocytes. Present results support the view that the synaptic transmission events supporting neuroendocrine signaling in the hypothalamus follow similar neuroglial compartmentation mechanisms to other types of cerebral sensorial or motor activations.

## Recapitulation

**Figure 7:**
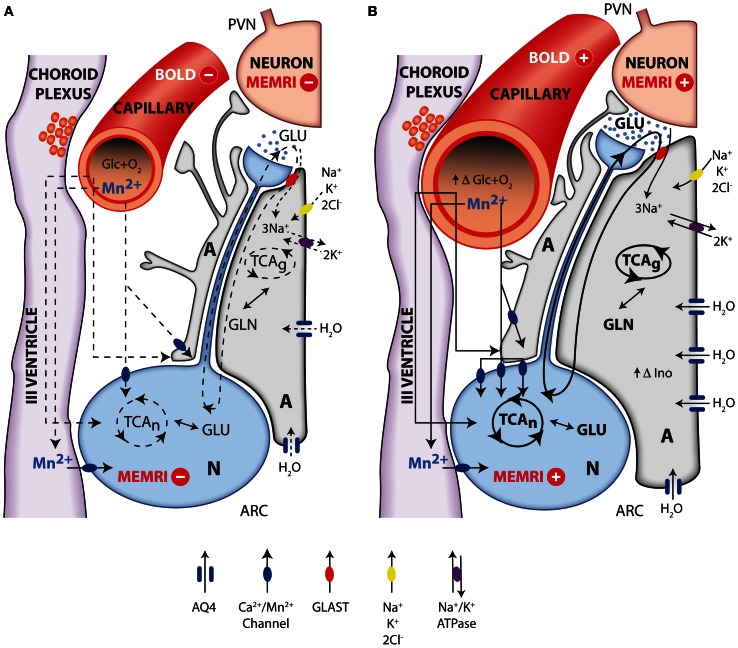
**Integrative interpretation of orexigenic activation in the hypothalamus as detected by MEMRI, BOLD, fDWI, and multinuclear magnetic resonance spectroscopy**. Neuroglial metabolic coupling mechanisms and volume changes in astrocytes and capillaries of the hypothalamus underlie the orexigenic response from resting **(A)** to activated **(B)** states. Briefly, during orexigenic neurotransmission, excess glutamate released to the synaptic cleft by AGRP/NPYY neurons (N, blue) is recaptured by surrounding astrocytes (A, gray), together with 3Na^+^. Intracellular sodium ions incorporated are extruded to the extracellular space, through the electrogenic Na^+^/K^+^ATPase, incorporating two intracellular K^+^ ions. Astrocytic glutamate produces glutamine, through glutamine synthase, which is later recaptured by the neurons to operate the glutamate-glutamine cycle. Additional K^+^ ions may be incorporated to the astrocyte through the Na^+^K^+^2Cl^−^ cotransporter, resulting in increased intracellular K^+^ concentrations and thus triggering an osmotically driven, aquaporin 4 (AQP4) mediated (blue channel), water transport, producing astrocytic swelling. Glucose and oxygen are delivered through capillary to astrocytes and neurons, and Mn^2+^ ions enter neurons and astrocytes through calcium channels. Under resting conditions **(A)**, oxidative metabolism and glutamatergic neurotransmission depict basal levels (dotted lines). Under activated orexigenic conditions **(B)**, oxidative metabolism and glutamatergic neurotransmission are increased (darker lines, increased glutamate in the synaptic cleft) as revealed by ^13^C HRMAS, resulting in augmented ionic and water trafficking to astrocytes (darker arrows, increased water influx through AQP4). The accumulated K^+^ in one synaptic astrocyte may be transferred to the neighboring astroglia, through gap junctions, providing a spreading mechanism for the swelling response through the astrocyte syncytium, becoming measurable by fDWI. Astrocytic volume changes are thought to underlie the increased hypothalamic myo-inostol content observed by ^1^H HRMAS. Glucose and oxygen demand increase (increasing capillary volume) during neuronal activation, and the ratio between deoxyhemoglobin and oxyhemoglobin changes causing the BOLD effect and the increased ^13^C labeling of glutamate and GABA. Neuronal accumulation of Mn^2+^ through voltage dependent calcium channels increase with neuronal activation (higher number of channels and darker lines), causing the MEMRI effect.

In summary, the evidence accumulated recently by MRI and MRS methodologies has contributed importantly to investigate the role of metabolic compartmentation during neuroendocrine regulation in the hypothalamus. Figure 7 provides an integrative interpretation on the role of neuroglial compartmentation during appetite regulation, as revealed by MEMRI, BOLD, DWI as well as by ^1^H and ^13^C NMR spectroscopy.

Briefly, the firing of orexigenic neurons involves voltage dependent Na^+^ and Ca^2+^ channels. Mn^2+^ substitutes Ca^2+^, accumulating in excited neurons during depolarization (Koretsky and Silva, 2004; Silva et al., 2004). In addition, glutamatergic neurotransmission is known to be associated to intracellular and intercellular astrocyte to astrocyte calcium waves transmitted through gap junctions (Jaffe, 2006, 2008). Mn^2+^ could thus accumulate in astrocytic networks as well. These astrocytic arrangements may reach millimeter sizes, becoming then detectable under conventional MRI resolution conditions. At present it is not clear which neuronal or astroglial mechanism is predominant, but it can be safely thought that both contribute to the observed MEMRI effect. Excess glutamate released to the orexigenic cleft under fasting or obese conditions, is recaptured by surrounding astrocytes, by Na^+^ dependent cotransport mainly through the GLAST/EAAT1 and GLT-1/EAAT2 transporters, in a 3Na^+^ per glutamate stoichiometry (Anderson and Swanson, 2000). The three sodium ions incorporated in this way, are extruded to the extracellular space, in exchange with two potassium ions entering the astrocytic interior through the electrogenic Na^+^/K^+^ ATPase (Glynn, 1993; Pellerin and Magistretti, 1994). The astrocytic ATP required for the operation of the Na^+^/K^+^ ATPase and glutamine synthesis during activation by fasting, is thought to be derived from increased glucose consumption and metabolism by oxidative and glycolytic pathways in neurons and astrocytes (Cerdan et al., 2006; Violante et al., 2009; Delgado et al., 2011). Indeed, orexigenic stimulation results in lactate accumulation in the hypothalamus and increased labeling of glutamate, glutamine and GABA from (1-^13^C) glucose as reflected by ^13^C HRMAS. This reveals that hypothalamic activation by fasting involves the excitation of both glutamatergic (activatory) and GABAergic (inhibitory) terminals, as expected for an hypothalamic feed-back loop mechanism (Figure 1). In addition, the increased metabolic demand in the hypothalamus induced by fasting, results in an increased microvascular blood flow and hemoglobin deoxygenation, a circumstance underlying the changes observed by BOLD and DWI imaging (Lizarbe et al., 2013).

The volume changes inferred in the hypothalamus during orexigenic activation merit further attention. The K^+^ ions accumulated in the extracellular space during the orexigenic action potentials, may enter surrounding astrocytes through stimulation of the Na^+^/K^+^/2Cl^−^ cotransporter (Hertz et al., 2007), a circumstance that might trigger concomitant water influx and astrocytic volume increase (Jayakumar and Norenberg, 2010), primarily-mediated through the highly abundant aquaporin AQP-4 of astrocytic membranes (Badaut et al., 2002). It should be noted here that increased K^+^ concentrations are known to be tightly coupled to neuronal activation, and have been detected using metallographic microscopic imaging approaches (Goldschmidt et al., 2004). In addition, EAAT1 glutamate sodium cotransporter has been shown to cause water influx together with glutamate transport (MacAulay et al., 2001) and the activation of the GABA receptors GABA_A_Rs has been recently proposed to be involved with cell volume regulation processes and water exchange in the brain (Cesetti et al., 2011). On these grounds, increased astrocytic volume may become an important determinant of the alterations in diffusion parameters observed by DWI. The osmotic swelling response associated to orexigenic stimulation, is proposed here to occur initially in the few astrocytes surrounding the activated orexigenic clefts (Figure 1), but can be rapidly extended, to a plethora of neighboring astrocytes, through the numerous interconnecting gap junctions of the network arrangement (Halassa and Haydon, 2010) as mentioned above for Mn^2+^ accumulations.

The volume changes inferred by DWI find an adequate support when considering the increases in hypothalamic *myo*-inositol levels detected by high field ^1^H MRS (Lei et al., 2010). The relative increases in osmolite content between the hypothalamus and other areas of the brain as detected by ^1^H MRS *in vivo*, indicate that volume regulation processes in the hypothalamus may play an important role in hypothalamic function.

In summary, the above sections indicate that MRI and MRS methodologies have provided important insight into the hypothalamic mechanisms underlying appetite regulation. Briefly, MEMRI approaches reveal neuroglial manganese accumulation, BOLD shows concomitant oxygen consumption and associated hemodynamic responses and DWI discloses water diffusion changes compatible with glial swelling. ^1^H and ^13^C MRS have revealed osmolite accumulation and increased glutamatergic and GABAergic neurotransmissions. Taken together, these results suggest an important role for hypothalamic metabolic compartmentation during appetite regulation, as in other cerebral activations. Naturally, these interpretations do not exclude additional contributions to MRI or MRS from other potential mechanisms.

Finally, the approaches reviewed here may provide a valuable tool to further investigate glutamatergic or GABAergic neurotransmissions in the hypothalamic control of global energy balance and in the development of improved treatments against feeding disorders, obesity, and diabetes. In addition, this arsenal of new methodologies may be easily extended to explore other hypothalamic functions, opening a new avenue in the research of hypothalamic performance *in vivo*.

### Conflict of interest statement

The authors declare that the research was conducted in the absence of any commercial or financial relationships that could be construed as a potential conflict of interest.

## Abbreviations

MRI: Magnetic Resonance Imaging
MRS: Magnetic Resonance Spectroscopy
MEMRI: Manganese-enhanced Magnetic Resonance Imaging
BOLD: Blood Oxygenation Level-dependent contrast
DWI: Diffusion-weighted Imaging
DIA: Dehydration-induced Anorexia.
